# Effect of Cusp-Overlap View Technique on the Occurrence of Post-Procedural New Conduction Disturbance and Permanent Pacemaker Implantation Following Transcatheter Aortic Valve Replacement Using Self-Expanding Prostheses

**DOI:** 10.3390/jcm15114009

**Published:** 2026-05-22

**Authors:** Mostafa Salem, Jakob Voran, Mohamed Salem, Rafael Rangel, Hatim Seoudy, Annika Strake, Georg Lutter, Johanne Frank, Derk Frank, Mohammed Saad

**Affiliations:** 1 Department of Internal Medicine III, Cardiology and Intensive Care, University Hospital Schleswig-Holstein, 24105 Kiel, Germany; 2 DZHK (German Center for Cardiovascular Research), Partner Site Hamburg/Kiel/Lübeck, 24105 Kiel, Germany; 3 Department of Congenital Heart Surgery and Congenital Heart Defects, Pediatric Heart Center, University Hospital Giessen and Marburg, 35392 Giessen, Germany; pd.dr.med.m.salem@gmail.com; 4 Department of Cardiovascular Surgery, University Clinical Center Schleswig-Holstein, 24105 Kiel, Germany

**Keywords:** aortic valve, transcatheter aortic valve replacement, cusp-overlap technique, pacemaker implantation

## Abstract

**Objective:** Self-expanding (SE) transcatheter aortic prostheses (THV) have been associated with an increased risk of new permanent pacemaker implantation (PPMI), particularly with deeper implantations in the left ventricular outflow tract (LVOT) that result in more atrioventricular conduction system damage, leading to higher rates of post-procedural conduction disturbances (CDs) and subsequently more PPMIs. The cusp-overlap technique (COT) is designed to provide better visualisation of the LVOT during implantation, aiming to achieve a shallower implantation depth (ID) and potentially reduce both post-procedural CDs and PPMIs. This study seeks to compare the traditional three-cusp coplanar view technique (3CT) with the newer COT in patients undergoing transcatheter aortic valve replacement (TAVR). **Methods:** From March 2018 to April 2020, a total of 586 patients underwent TAVR at the university clinic in Kiel. Among them, 226 patients who received SE prostheses were included in the study. After applying exclusion criteria, a final cohort of 203 patients was analysed. Of these, 106 patients underwent TAVR using the COT, while 97 patients underwent TAVR using the 3CT. The primary endpoints of the study were the occurrence of new CD and PPMI within 30 days post-procedure. Secondary endpoints included various post-TAVR events as defined by the Valve Academic Research Consortium 3 (VARC-3) safety criteria. A specific focus was placed on assessing the risk of high valve implantation according to VARC-3 criteria, specifically paravalvular insufficiency, valve embolisation, and coronary occlusion. Statistical analysis was conducted to compare outcomes between the COT and 3CT groups. **Results:** Implantation depths were significantly lower in the COT group compared to the 3CT group, with ID values from the NCC and LCC being 2.7 mm (±1.5) and 2.8 mm (±1.5) for the COT, and 5.4 mm (±3) and 6.6 mm (±2.6) for the 3CT (*p* < 0.001 for both). The incidence of high-grade CD, particularly Atrioventricular Block (AVB) II and III, was significantly higher in the 3CT group (26.8%) compared to the COT group (13.2%) (*p* = 0.023). The overall 30-day PPMI rate was 18.2% (n = 37), with a significant difference between the COT and 3CT groups (12.2% vs. 24.7%, *p* = 0.021). The primary indication for PPMI was permanent high-grade AVB occurring during or after TAVR, accounting for 95% of cases. No cases of TAVR embolisation, acute coronary occlusion or related syndromes were observed within the first 30 days post-procedure. There were no significant differences in 30-day mortality or post-procedural paravalvular insufficiency between the groups. In multivariable logistic regression analysis, the COT remained independently associated with lower odds of new post-procedural CD after adjustment for prior right bundle branch block (RBBB), prior first-degree AVB, predilatation, valve size and coronary artery disease (odds ratio [OR] 0.45, 95% confidence interval [CI] 0.24–0.82, *p* = 0.009). For 30-day PPMI, the cusp-overlap technique demonstrated a borderline association with lower adjusted odds (OR 0.46, 95% CI 0.20–1.02, *p* = 0.057), while prior RBBB was independently associated with increased PPMI risk (OR 3.54, 95% CI 1.22–10.28, *p* = 0.020). **Conclusions:** The COT was associated with shallower implantation depth and lower rates of new post-procedural CD after multivariable adjustment. The association with reduced 30-day PPMI remained directionally consistent but was borderline after adjustment. These findings support the potential value of COT as a procedural strategy to reduce conduction-related complications after TAVR with self-expanding prostheses.

## 1. Introduction

Transcatheter aortic valve replacement (TAVR) is now widely accepted for managing severe aortic stenosis (AS) in various risk categories. Before, it was limited only to high-risk or inoperable patients [1]. The indication for TAVR expanded to include intermediate- [2,3] and low-risk elderly patients [4,5,6,7]. Despite improvements in procedural techniques and device design, complications like new conduction disturbances (CDs) leading to permanent pacemaker implantation (PPMI) continue to be a concern, with an incidence ranging from 3.4% to 25.9% [6,8]. PPMI has been associated with more extended hospital stays, increased costs (accounting for 25% of TAVR procedure expenses) [9], and worse long-term prognosis [10,11,12,13,14]. Notable factors predicting post-TAVR conduction issues [6] include pre-existing right bundle branch block, use of self-expanding prostheses (SE), balloon post- and pre-dilation, and a lower valve implantation depth (ID) [6,15,16,17,18]. As deeper implantations interfere with the conduction system and consequently produce CD, achieving optimal ID is crucial to reduce those complications. By overlapping the right (RCC) and left coronary cusps (LCC) and isolating the non-coronary cusp (NCC), the cusp-overlap technique (COT) offers enhanced visualisation and accurate positioning. In this study, we compared 30-day outcome rates of new-onset of persistent CD, PPMI rates and the importance of reducing the ID on these outcomes between patients undergoing TAVR with the SE Evolut R™ (ER) and Evolut PRO™ (EP) (Medtronic) transcatheter heart valves (THVs) using the conventional three-cusp coplanar view technique (3CT) and the more recently described right/left cusp-overlap view (COT).

## 2. Methods

### 2.1. Study Population and Design

This retrospective observational study took place at a single centre and included all patients who had transcatheter aortic valve replacement for severe native aortic valve disease at the University Heart Center Kiel from March 2018 to April 2020. Only patients treated with new-generation self-expanding transcatheter heart valves, specifically CoreValve Evolut R™ and Evolut PRO™ (Medtronic, Minneapolis, MN, USA), were included.

Researchers collected clinical, procedural, and follow-up data from the hospital’s electronic systems—Orbis^®^ (Dedalus HealthCare GmbH, Bonn, Germany), enaio^®^ (OPTIMAL SYSTEMS GmbH, Berlin, Germany), and Meona^®^ (Mesalvo Freiburg GmbH, Freiburg im Breisgau, Germany)—and from archived patient records. The data included patients’ baseline demographics, comorbidities, details of in-hospital treatment, procedural information, and follow-up outcomes.

The multidisciplinary heart team at the institution decided on treatment based on current clinical guidelines. They referred patients for surgical aortic valve replacement, transcatheter aortic valve replacement, or conservative medical management according to each patient’s anatomy, clinical status, and procedural factors. Experienced operators at a high-volume heart valve centre performed or directly supervised all transcatheter aortic valve replacement procedures.

### 2.2. Inclusive and Exclusive Criteria

Consecutive patients undergoing transfemoral transcatheter aortic valve replacement (TAVR) with new-generation self-expanding Evolut prostheses were assessed for eligibility. Exclusion criteria included age under 18 years, inability to provide informed consent, non-transfemoral access, valve-in-valve procedures, bicuspid aortic valve morphology, and presence of a permanent pacemaker before the index procedure. Among 226 patients screened, 203 met the predefined criteria and were included in the final study cohort.

#### Ethics Statement

The study protocol received approval from the local ethics committee of the University Hospital Schleswig-Holstein, Campus Kiel, Germany (Ethics Committee of the Medical Faculty, Christian-Albrechts-University of Kiel; approval no. D 506/22). The study adhered to the principles outlined in the Declaration of Helsinki. Written informed consent was obtained from all participants or their legal representatives.

### 2.3. Transcatheter Aortic Valve Replacement Procedure (TAVR) [19]

**Figure 1:**
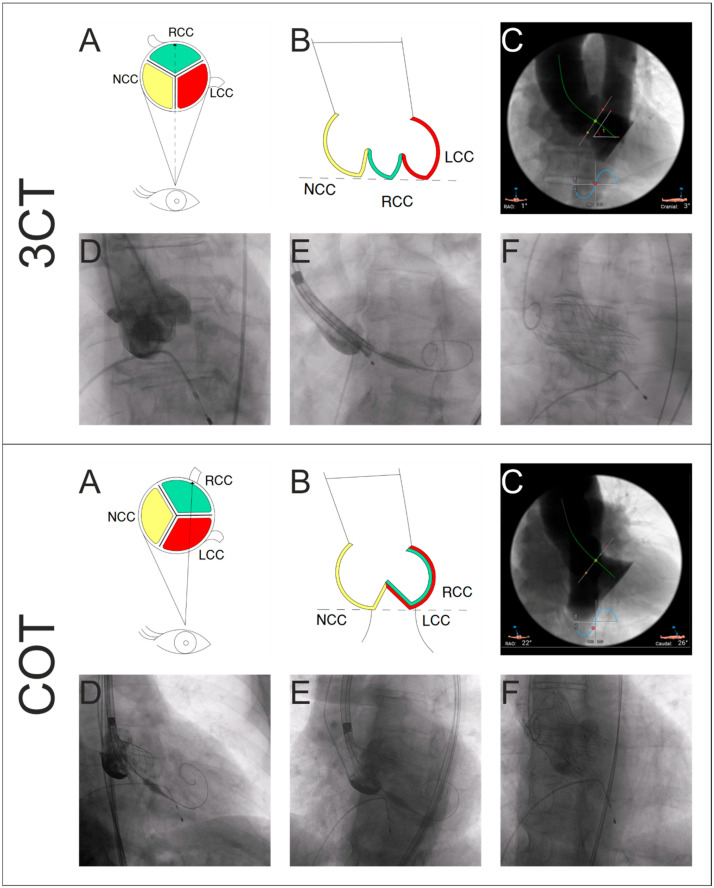
Comparison of implantation techniques 3CT and COT. The upper panel shows the 3CT implantation technique, the lower panel the COT implantation technique. (**A**) schematic drawing of pre-operative axial reconstruction, (**B**) schematic drawing of the aorta during TAVR procedure using fluoroscopy, (**C**) projection in 3mensio, (**D**) aortography during TAVR, (**E**) TAVR implantation, (**F**) final result without parallaxb. The eye image and the indicator line ending with the arrow indicate the direction of projection in fluoroscopy. In 3CT implantation technique, during the fluoroscopy to guide the implantation, the RCC is positioned between the NCC and LCC. In COT, the RCC and LCC overlap in fluoroscopy. 3CT: three-cusp coplanar projection technique; COT: cusp-overlap coplanar projection technique; LCC (red): left coronary cusp; NCC (yellow): non-coronary cusp; RCC (green): right coronary cusp.

Valve sizing followed manufacturer recommendations, using pre-procedural CT to assess the annulus, LVOT dimensions, and calcification. In the 3CT group, valve implantation used the conventional three-cusp coplanar projection, planned on CT, confirmed angiographically, and shown in Figure 1. In the COT group [20], the projection overlapped the right and left coronary cusps and isolated the non-coronary cusp, as shown in Figure 1, with angiographic confirmation before deployment. Valve positioning, pacing, repositioning, predilatation, and postdilatation were performed at the operator’s discretion and according to institutional practice. Implantation depth was assessed in the procedural projection. All patients received 12-lead electrocardiography before TAVR, 24 h post-procedure, before discharge, and at 30-day follow-up. PPMI was considered for patients with persistent complete or high-grade atrioventricular block during or after TAVR without spontaneous recovery, especially with pre-existing conduction abnormalities such as right bundle branch block or first-degree atrioventricular block. All procedures were performed or supervised by experienced TAVR operators at a high-volume tertiary heart valve centre.

### 2.4. Study Endpoints

The primary endpoint of the study was to assess the 30-day post-procedural occurrence and incidence of new CD and PPMI. The secondary endpoints included evaluating various criteria outlined in the Valve Academic Research Consortium 3 (VARC-3) safety definitions. These criteria encompassed post-TAVR events such as aortic regurgitation, all-cause death, myocardial infarction, stroke, vascular complications, and major/minor bleeding. Additionally, there was a particular focus on assessing the risk of high valve implantation according to VARC-3 criteria, explicitly paravalvular insufficiency, valve embolisation, and coronary occlusion.

### 2.5. Statistical Analysis

We assessed the normality of continuous variables using the Kolmogorov–Smirnov test. Normally distributed variables were presented as mean ± standard deviation and compared using *t*-tests. Non-normally distributed variables were compared using the Wilcoxon rank test. Categorical variables were shown as counts (percentages) and compared using chi-square tests. A *p*-value < 0.05 at the 95% confidence level was considered statistically significant for all tests.

Univariable and multivariable binary logistic regression analyses were conducted to identify predictors of 30-day permanent pacemaker implantation and new post-procedural conduction disturbances. The primary multivariable models incorporated implantation technique, prior right bundle branch block, prior first-degree atrioventricular block, predilatation, valve size, and coronary artery disease. Variable selection was guided by clinical relevance, established predictors of post-transcatheter aortic valve replacement (TAVR) conduction disturbance, and baseline group imbalances. Results are reported as odds ratios (ORs) with 95% confidence intervals (CIs). Due to the limited number of outcome events, the models were constrained to minimise the risk of overfitting. Statistical significance was defined as a two-sided *p*-value less than 0.05.

All analyses were performed using Analyse-it Version 6.15 software as an Add-on to Microsoft Excel (Analyse-it Software Ltd., Leeds, UK), integrated with Microsoft Excel and IBM SPSS Statistics, version 31 (IBM Corp., Armonk, NY, USA).

## 3. Results

### 3.1. Baseline Characteristics of the Study Population (Table 1)

Between March 2018 and April 2020, our centre performed 586 TAVR procedures. Among these, 226 patients were implanted with SE prostheses. However, only 203 of these patients were included in the current analysis, as several were excluded due to pre-existing pacemaker implants or lack of consent. Of the included patients, 106 underwent TAVR with the COT implantation view, while the remaining 97 received TAVR using 3CT.

**Table 1:** Baseline characteristics.

Characteristic	3CT, *n* = 97 ^1^	COT, *n* = 106 ^1^	*p*-Value ^2^
Age	81.8 (±5.5)	82.2 (±5.6)	>0.90
female	38/97 (39.1%)	60/106 (56.6%)	0.01
BMI [kg/m^2^]	27 (±5.02)	27.07 (±5.05)	>0.90
Diabetes	49/97 (50.5%)	50/106 (47.2%)	0.63
Dyslipidaemia	59/97 (60.8%)	68/106 (64.1%)	0.62
Hypertension	93/97 (95.9%)	101/106 (95.3%)	0.84
Atrial fibrillation or flutter	42/97 (43.3%)	55/106 (51.9%)	0.22
CAD	50/97 (51.5%)	70/106 (66%)	0.04
PAD	16/97 (16.5%)	16/106 (15.1%)	0.78
COPD	8/97 (8.24%)	15/106 (14.2%)	0.18
Previous cardiac surgery	19/97 (19.6%)	30/106 (28.3%)	0.15
Prior RBBB	8/97 (8.2%)	12/106 (11.3%)	0.46
Prior LBBB	9/97 (9.3%)	10/106 (9.4%)	0.79
Prior AVB I	29/97 (29.9%)	26/106 (24.5%)	0.39
Prior LAHB	6/97 (6.1%)	7/106 (6.6%)	0.90
NYHA class			0.02
I	7/96 (7.3%)	4/102 (3.9%)	
II	20/96 (20.8%)	42/102 (41.1%)	
III	56/96 (58.3%)	46/102 (45.1%)	
IV	13/96 (13.5%)	10/102 (9.2%)	
STS-Score [%]	4.2 (±4)	4.7 (±3.3)	0.38
Euroscore II	7.3 (±6.5)	6.1 (±6.3)	0.21
Ejection fraction	50.4 (±11.6)	51.6 (±9.2)	0.43
Ejection fraction category			0.25
I	60/97 (61.8%)	66/106 (62.3%)	
II	13/97 (13.4%)	23/106 (21.7%)	
III	14/97 (14.4%)	9/106 (8.5%)	
IV	10/97 (10.3%)	8/106 (7.5%)	
AV-MPG	41.37 (±15.7)	42.4 (±16.5)	0.66
AV-PPG	66.93 (±20.72)	70.69 (±18.93)	0.60
sPAP	45.28 (±13.74)	45.91 (±14.58)	0.80
Aortic regurgitation category			0.40
I	84/97 (86.6%)	90/106 (84.9%)	
II	12/97 (12.3%)	15/106 (14.2%)	
III	1/97 (1.03%)	0/106 (0%)	
IV	0/97 (0.00%)	1/106 (0.94%)	
AVA [cm^2^]	0.73 (±0.17)	0.74 (±0.16)	0.77

BMI: Body-mass index, CAD: coronary artery disease, COPD: Chronic obstructive pulmonary disease, LBBB: left bundle branch block, RBBB: right bundle branch block, AVB: atrioventricular block, LAHB: left anterior hemiblock, PAD: peripheral arterial occlusive disease, NYHA: New York Heart Association Classification, STS-Score: Society of Thoracic Surgeons scores, AV-MPG: aortic valve mean pressure gradient, AV-PPG: aortic valve peak pressure gradient, sPAP: systolic pulmonary artery pressure, AVA: aortic valve area. ^1^ Mean (SD); n/N (%); ^2^ Wilcoxon rank sum test; Pearson’s Chi-squared test; Fisher’s exact test.

Table 1 summarises the baseline characteristics of the study population. On average, patients were 82 years old (±6). Ninety-eight patients were female (48.2%), and there was a similar distribution of cardiovascular risk factors and relevant past medical history between groups.

The majority of patients were electively admitted with intermediate surgical risk, as evidenced by a mean EuroSCORE II of 6.1 (±6.3) for the COT and 7.3 (±6.5) for the 3CT (*p*-value = 0.21) and a Society of Thoracic Surgeons score of 4.7 (±3.3) for the COT and 4.2 (±4) for the 3CT (*p*-value = 0.38).

When comparing the two groups, the COT group showed a similar prevalence of hypertension (95.3% vs. 95.9%, *p*-value = 0.84), more cases of New York Heart Association functional class III or IV (71.8% vs. 54.3%, *p*-value = 0.02), and a nearly identical baseline left ventricular ejection fraction (51.6% (±9.2) vs. 50.4% (±11.6), *p*-value = 0.430) compared to the 3CT group.

The primary underlying indication for TAVR in all cases was severe native aortic valve stenosis (100%), characterised by a mean aortic valve gradient of 42.4 mmHg (±16.5) and aortic opening area of 0.74 (±0.16) for the COT group, and 41.37 mmHg (±15.7) and 0.73 (±0.17) respectively for the 3CT group (*p*-value = 0.66 and 0.77 respectively).

Overall, baseline conduction abnormalities were balanced between groups. Prior right bundle branch block (RBBB) was identified in 8 of 97 patients (8.2%) in the 3CT group and 12 of 106 patients (11.3%) in the COT group (*p* = 0.46). Prior left bundle branch block (LBBB) was observed in 9 of 97 patients (9.3%) and 10 of 106 patients (9.4%), respectively (*p* = 0.79). First-degree atrioventricular block (AVB) was present in 29 of 97 patients (29.9%) in the 3CT group and 26 of 106 patients (24.5%) in the COT group (*p* = 0.390). Left anterior hemiblock was documented in 6 of 97 patients (6.1%) and in 7 of 106 patients (6.6%) (*p* = 0.90).

### 3.2. Procedural Data, Hospitalisation, and 30-Day Outcomes (Table 2)

All patients underwent TAVR via common femoral artery access using the new-generation SE prostheses, CoreValve Evolut R™ (ER) and Evolut PRO™ (EP) valves. Predilatation was required in 43.6% of the COT group and 64.5% of the 3CT group, with postdilatation needed in 34.7% and 30.1%, respectively. Only one patient required a second valve due to severe aortic regurgitation, and there were no cases of TAVR embolisation ‘pop-out’ in either group. The mean procedure duration was 58.3 (±20.3) minutes for the COT group and 57.9 (±24.5) minutes for the 3CT group (*p*-value = 0.88).

**Figure 2:**
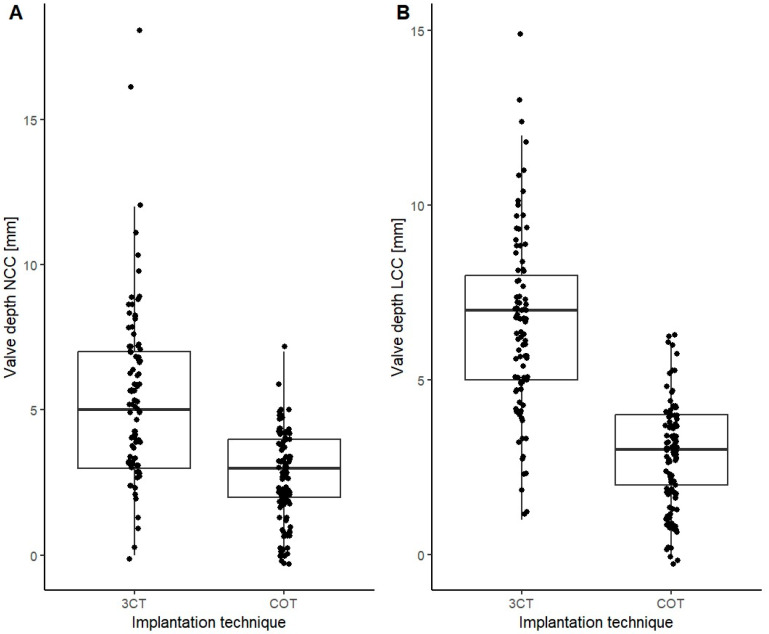
Valve depth by implantation technique. (**A**) Comparison of valve implantation depth according to the position of the NCC. (**B**) Comparison of valve implantation depth according to the position of the LCC. 3CT: three-cusp coplanar projection technique; COT: cusp-overlap coplanar projection technique; LCC: left coronary cusp; NCC: non-coronary cusp.

The implantation depths (IDs) were significantly lower in the COT group compared to the 3CT group. The IDs according to NCC and LCC for the COT group were 2.7 mm (±1.5) and 2.8 mm (±1.5), respectively, while for the 3CT group, they were 5.4 mm (±3) and 6.6 mm (±2.6), respectively (*p*-value ≤ 0.001 for both). Figure 2 depicts these findings.

**Table 2:** Peri- and post-procedural data and results.

Characteristic	3CT, *n* = 97 ^1^	COT, *n* = 106 ^1^	*p*-Value ^2^
New conduction disturbance	51/97 (52.5%)	34/106 (32.0%)	0.003
Type new conduction disturbance			0.06
AVB I	5/97 (5.2%)	6/106 (5.6%)	0.87
AVB II	4/97 (4.1%)	1/106 (0.9%)	0.34
AVB III	22/97 (22.7%)	13/106 (12.3%)	0.05
LBBB	13/97 (13.4%)	8/106 (7.5%)	0.17
RBBB	1/97 (1.0%)	0/106 (0.0%)	0.22
SSS	3/97 (3.1%)	1/106 (0.94%)	0.26
AF	3/97 (3.1%)	4/106 (3.8%)	0.79
VT	0/97 (0.0%)	1/106 (0.94%)	0.25
High-grade AVB (AVB II + AVB III)	26/97 (26.8%)	14/106 (13.2%)	0.02
High-grade AVB + LBBB	39/97 (40.2%)	22/106 (20.7%)	0.002
New pacemaker implantation	24/97 (24.7%)	13/106 (12.2%)	0.02
Type of pacemaker			0.14
VVI-PM	5/24 (20.8%)	2/13 (15.3%)	0.20
DDD-PM	14/24 (58.3%)	9/13 (69.2%)	0.18
CRT-P	5/24 (20.8%)	2/13 (15.3%)	0.20
Valve depth NCC [mm]	5.4 (±3)	2.7 (±1.5)	<0.001
Valve depth LCC [mm]	6.6 (±2.6)	2.8 (±1.5)	<0.001
Implantation attempts			0.55
1	79/97 (81.4%)	89/106 (84%)	
2	13/97 (13.4%)	14/106 (13.2%)	
3	3/97 (3.1%)	3/106 (2.8%)	
4	1/97 (1.03%)	0/106 (0.0%)	
5	1/97 (1.03%)	0/106 (0.0%)	
Valve size [mm]			0.88
23	2/97 (2.0%)	4/106 (3.8%)	
26	22/97 (22.7%)	25/106 (23.6%)	
29	46/97 (47.4%)	50/106 (47.2%)	
34	27/97 (27.8%)	27/106 (25.5%)	
Valve type			0.30
Evolut™ PRO	18/97 (18.6%)	14/106 (13.2%)	
Evolut™ R	79/97 (81.4%)	92/106 (86.8%)	
Procedure Duration [min]	57.9 (±24.5)	58.3 (±20.3)	0.88
Contrast agent used [ml]	92.7 (±35)	82 (±27.3)	0.02
Post-implantation aortic regurgitation category			0.48
0	67/97 (69%)	80/106 (75.5%)	
I	22/97 (22.7%)	21/106 (19.8%)	
II	8/97 (8.2%)	5/106 (4.7%)	
Stroke	1/97 (1.03%)	3/106 (2.8%)	0.34
Major vascular access site complication	0/97 (0.0%)	0/106 (0.0%)	
Minor vascular access site complication	0/97 (0.0%)	0/106 (0.0%)	

AVB: atrioventricular block, LBBB: left bundle branch block, RBBB: right bundle branch block, SSS: sick sinus syndrome, AF: atrial fibrillation, VT: ventricular tachycardia, VVI-PM: one chamber pacemaker, DDD-PM: two chambers pacemaker, CRT-P: Cardiac resynchronisation therapy with a pacemaker, S-ICD: Subcutaneous implantable cardioverter defibrillator. ^1^ n/N (%); Mean (SD); ^2^ Pearson’s Chi-squared test; Fisher’s exact test; Wilcoxon rank sum test.

The incidence of high-grade CD, including AVB II and III, was significantly higher in the 3CT group than in the COT group, with percentages of 26.8% (26 patients) and 13.2% (14 patients), respectively (*p*-value = 0.02).

There were no cases of acute coronary occlusion or acute coronary syndromes in the first 30 days. Intraprocedural/periprocedural new-onset LBBB was detected in 21 patients across both groups, with a higher rate in the 3CT group (13.4%; 13 patients). In comparison, the COT group had only 7.5% (8 patients) with new LBBB (*p*-value = 0.17).

**Figure 3:**
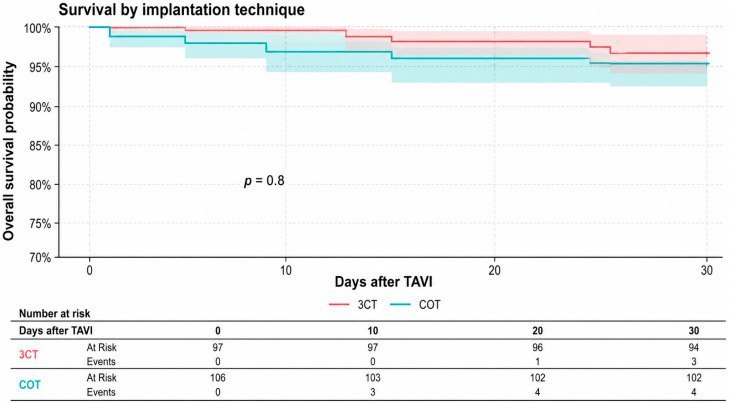
Mortality comparison between 3CT and COT.

Four patients (3.7%) in the COT group and three patients (3.1%) in the 3CT group had 30-day mortality from all causes, according to the VARC-3 definition (*p*-value = 0.21). In the COT group, the first patient died on the day of TAVR due to cardiogenic/haemorrhagic shock with pericardial effusion after TAVR. The second patient passed away on the 5th post-procedural day due to cardiogenic shock with severe PVL after TAVR. The third patient died on the 9th post-procedural day after discharge following resuscitation for pulseless electrical activity (PEA) without a clear determination of the exact cause of death. The fourth patient died on the 15th post-procedural day after discharge without a clear determination of the cause of death. In the 3CT group, the first patient died on the 13th post-procedural day from mixed septic/cardiogenic shock in the surgical intensive care unit after emergency thoracotomy for perforation of the right ventricle during TAVR. The second patient passed away after discharge on the 24th post-procedural day without a clear determination of the cause of death. The third patient died on the 25th post-procedural day after discharge following readmission for pleural puncture, resulting in iatrogenic haemothorax, leading to death from mixed haemorrhagic, respiratory, and cardiogenic shock (Figure 3).

The 30-day permanent pacemaker implantation (PPMI) rate for all patients in this study was 18.2% (n = 37), with a significant difference between the COT and 3CT groups (12.2% vs. 24.7%, *p*-value 0.02). The main underlying reason for PPMI was permanent high-grade atrioventricular block (AVB II and AVB III) during or after 24 h post-TAVR, accounting for 95% of cases.

In the primary multivariable binary logistic regression model adjusted for the implantation technique, prior RBBB, prior first-degree AVB, predilatation, valve size and coronary artery disease. Patients treated with the COT were less likely to require PPMI within 30 days, even after adjustment for other relevant risk factors. However, this association did not achieve conventional statistical significance (OR 0.46, 95% CI 0.20–1.02, *p* = 0.06). Prior RBBB was independently associated with a higher risk of 30-day PPMI (OR 3.54, 95% CI 1.22–10.28, *p* = 0.02). In contrast, prior first-degree AVB exhibited a non-significant trend toward increased pacemaker risk (OR 1.94, 95% CI 0.88–4.26, *p* = 0.10) (Table 3).

In a second multivariable model, which used new post-procedural CD as the dependent variable and adjusted for the same covariates, the COT remained independently associated with lower odds of new CD (OR 0.45, 95% CI 0.24–0.82, *p* = 0.01). In this model, prior RBBB, prior first-degree AVB, predilatation, valve size, and coronary artery disease were not independently associated with new CD (Table 4).

Additionally, a significant difference was observed in contrast agent use: the COT group utilised 82 mL (±27.3), compared with the 3CT group utilising 92.7 mL (±35), resulting in a *p*-value of 0.017.

## 4. Discussion

The COT implantation technique has become widely used in TAVR procedures worldwide. This study aimed to evaluate the safety and efficacy of COT in SE prostheses compared to 3CT. Our findings emphasise several important points: The COT strategy is considered safe, as it achieves a higher implantation depth without reaching the AV-conduction system as deeply as in the 3CT approach, resulting in reduced contact with and damage to it. This finding was associated with a significant reduction in overall 30-day CD, particularly high-grade atrioventricular CD. The unadjusted 30-day PPMI rate was also lower in the COT group, while the adjusted association with PPMI remained directionally favourable but did not reach statistical significance.

The multivariable analyses further support the association between COT and reduced conduction-related complications. After adjustment for baseline conduction abnormalities, predilatation, valve size and coronary artery disease, the COT remained independently associated with lower odds of new post-procedural CD. For 30-day PPMI, the direction of effect was consistent with the unadjusted analysis, although the adjusted association was borderline and did not reach conventional statistical significance. These findings indicate that the COT is strongly associated with fewer new CDs, while the reduction in pacemaker implantation may be influenced by additional baseline and procedural factors. Prior RBBB remained independently associated with pacemaker implantation, supporting its established role as a major risk marker for pacemaker requirement after TAVR.

These findings have important clinical implications, as they demonstrate a significant reduction in new CD and a lower unadjusted pacemaker implantation rate in the COT group. However, the adjusted association between the COT and 30-day PPMI was borderline; therefore, the pacemaker-related results should be interpreted with caution.

This reduction in pacemaker implantation rates can improve patient outcomes and reduce the procedure’s potential risks.

Compared with the standard 3CT approach, the COT did not increase the risk of other complications, as defined by VARC-3. Notably, the COT approach remained feasible without increasing procedural complexity. This suggests that utilising the COT strategy may provide a safe alternative with similar outcomes and without introducing additional challenges during the TAVR procedure [3,5,21].

On the other hand, the COT approach utilised a significantly lower volume of contrast agent than the 3CT technique. This reduction in contrast agent volume can help minimise potential side effects associated with higher volumes during the TAVR procedure. By reducing the amount of contrast agents used, the COT approach may improve patient safety and outcomes.

Both the COT and 3CT techniques utilise the same materials and steps to release the valve, with no differences in procedures or materials. The only distinction lies in the modification of the working projection. Complications of shallower implantation, such as TAVR prosthesis embolisation ‘pop-out’, residual aortic regurgitation, coronary occlusions, and conversion to open-heart surgery, were rare occurrences. Additionally, there was almost no significant difference between the two groups in terms of 30-day mortality rates.

Given the standardisation of preprocedural measurements using Multislice Computed Tomography (MSCT) planning, incorporating the COT should not pose significant challenges during preprocedural assessment. In our clinical experience, implementing the COT did not increase the complexity of the established TAVR protocol. This observation is reinforced by the lack of noticeable differences in procedure duration (cut-to-stitches time) between the two study groups.

It is widely acknowledged that baseline conduction disturbances, such as pre-existing RBBB, AVB I, and left anterior hemiblock, are the most pertinent and unmodifiable independent predictors of PPMI after TAVR. Conversely, other robust predictors, such as predilatation, THV type, and prosthesis ID, are amenable to modification. Prudent management of these factors can effectively mitigate the risk of PPMI. For instance, SE prosthesis has been correlated with a 2.5-fold heightened risk of PPMI [22,23]. A recent meta-analysis encompassing the latest-generation prostheses revealed PPMI rates ranging from 14.7% to 26.7% for SE prostheses, compared with 4% to 24% for balloon-expandable valves [24,25]. Despite advancements in THV design and manufacturing, a significant clinical need remains to address this issue.

In a comprehensive meta-analysis by Yujing Chen, 3647 subjects from 11 studies were included. Among them, 1453 underwent SE TAVR using the COT and 2194 using the 3CT. The pooled analysis revealed that the cumulative incidence of PPMI was 9.3% in the COT group and 18.9% in the 3CT group. The use of the COT was associated with a significant reduction in the risk of PPMI (random-effects OR: 0.49, 95% CI: 0.36–0.66, *p*-value ≤ 0.001). Additionally, the COT group achieved a shallower implantation depth than the 3CT group (SMD = −0.324, 95% CI: −0.469 to −0.180). There were no significant differences between the two groups in second-valve implantation, prosthesis pop-out, fluoroscopic time, postoperative incidence of LBBB, mortality, stroke, moderate/severe paravalvular leakage, mean gradient, or length of hospital stay. However, radiation doses were higher in the COT group (*p*-value ≤ 0.001). In conclusion, this meta-analysis found that, in SE TAVR procedures, utilising the COT was associated with a lower risk of PPMI than the standard 3CT. In comparison with this study, our results align with all our findings except for the numerical value of LBBB incidence; in our study, the 3CT had a statistically insignificant, numerically higher LBBB incidence than the COT.

Despite some data suggesting a higher late mortality rate after TAVR in patients with newly acquired persistent LBBB, especially those with QRS durations over 160 milliseconds, it is important to emphasise that isolated LBBB is not an indication for PPMI [26]. In our study, there was no significant difference in the 30-day mortality rate between the two groups, as mentioned above. It is worth noting that our follow-up period in this study was limited to 30 days, so any subsequent mortality outcomes were not considered.

The primary focus of our investigation was to explore strategies for reducing conduction disturbances associated with SE prostheses. The proposed solution involves achieving a higher implantation position to minimise damage to the conduction system. However, implanting the prosthesis 5–7 mm below the aortic annulus increases the risk of PPMI. On the other hand, a higher deployment position can help decrease this risk. Conversely, an implantation depth (ID) of less than 3 mm may increase the risk of paravalvular regurgitation and coronary artery occlusion, as well as introduce the risk of valve embolisation [27]. Considering these factors, the manufacturer recommends maintaining an ID of 3–5 mm below the aortic annulus to optimise procedural outcomes and minimise complications.

Other studies have shown that membranous septal (MS) length can serve as an anatomical indicator of the distance between the aortic annulus and the bundle of HIS. Moreover, it is inversely associated with the risk of conduction system abnormalities following TAVR [28]. Additionally, it is recommended to include pre-procedural measurements of MS length to help determine the threshold for ID into the LVOT, facilitating better visualisation through the COT. Typically, the COT is obtained using the right anterior oblique (RAO) view with caudal angulation, providing optimal visualisation of the LVOT. This view often shows an elongated LVOT compared with the left anterior oblique (LAO) view, as demonstrated by MSCT images [19,29,30].

## 5. Limitations

Several limitations should be acknowledged. First, this was a retrospective, single-centre observational study with a limited sample size. Although multivariable adjustment was performed, the number of PPMI events remained low and residual confounding cannot be excluded particularly given baseline imbalances in coronary artery disease and NYHA functional class. Consequently, the regression analyses should be considered exploratory and hypothesis-generating. Second, implantation depth measurements may not be fully comparable between groups as they were obtained using different fluoroscopic projections in the COT and 3CT groups. Third, since the COT was introduced later in the study period, a potential learning-curve or temporal bias cannot be ruled out entirely.

## 6. Conclusions

In this retrospective single-centre cohort of patients undergoing TAVR with self-expanding Evolut prostheses, the cusp-overlap technique (COT) was associated with a shallower implantation depth and lower rates of new post-procedural conduction disturbances (CDs) compared to the three-cusp coplanar technique (3CT). The association with reduced 30-day permanent pacemaker implantation (PPMI) persisted after multivariable adjustment, although the association was borderline significant. No increase in relevant procedural complications, such as valve embolisation, acute coronary occlusion, or significant paravalvular regurgitation, was observed. These findings suggest that the COT may be a valuable procedural strategy to reduce conduction-related complications; however, prospective studies are required to confirm its independent effect on pacemaker implantation.

## Figures and Tables

**Table 3:** Multivariable logistic regression analysis for predictors of 30-day permanent pacemaker implantation.

Variable	OR	95% CI	*p*-Value
COT	0.46	0.20–1.02	0.06
Prior RBBB	3.54	1.22–10.28	0.02
Prior AVB I	1.94	0.88–4.26	0.10
Predilatation	1.19	0.52–2.74	0.68
Valve size	1.00	0.88–1.12	0.94
CAD	0.69	0.32–1.48	0.34

OR: odds ratio; CI: confidence interval; RBBB: right bundle branch block; AVB: atrioventricular block; CAD: coronary artery disease; COT: cusp-overlap technique.

**Table 4:** Multivariable logistic regression analysis for predictors of new post-procedural conduction disturbance.

Variable	OR	95% CI	*p*-Value
COT	0.45	0.24–0.82	0.009
Prior RBBB	1.40	0.53–3.70	0.50
Prior AVB I	1.23	0.64–2.35	0.53
Predilatation	0.96	0.49–1.89	0.91
Valve size	0.97	0.88–1.06	0.52
CAD	0.59	0.33–1.06	0.08

OR: odds ratio; CI: confidence interval; RBBB: right bundle branch block; AVB: atrioventricular block; CAD: coronary artery disease; COT: cusp-overlap technique.

## Data Availability

The datasets generated and/or analyzed during the current study are not publicly available due to patient privacy, confidentiality, and ethical restrictions. Data may be available from the corresponding author upon reasonable request, provided that the request is scientifically justified and approved by the responsible institutional ethics committee.

## Abbreviations

AS	aortic stenosis
CD	conduction disturbance
COT	cusp-overlap coplanar projection technique
ID	implantation depth
LCC	left coronary cusp
LVOT	left ventricle outflow tract
NCC	non-coronary cusp
RCC	right coronary cusp
SE	self-expanding
TAVR	transcatheter aortic valve replacement
THV	transcatheter heart valves, transcatheter aortic prostheses
3CT	three-cusp coplanar view technique
